# Unravelling the Molecular Regulation Mechanisms of Slow Ripening Trait in *Prunus persica*

**DOI:** 10.3390/plants10112380

**Published:** 2021-11-05

**Authors:** Gerardo Núñez-Lillo, Lissette Ulloa-Zepeda, Catalina Pavez, Anibal Riveros, Francisca Blanco-Herrera, Reinaldo Campos-Vargas, Romina Pedreschi, Claudio Meneses

**Affiliations:** 1 Escuela de Agronomía, Facultad de Ciencias Agronómicas y de los Alimentos, Pontificia Universidad Católica de Valparaíso, Calle San Francisco s/n, La Palma, Quillota 2260000, Chile; gera.nunez.lillo@gmail.com (G.N.-L.); romina.pedreschi@pucv.cl (R.P.); 2 Centro de Biotecnología Vegetal, Facultad de Ciencias de la Vida, Universidad Andres Bello, Santiago 8370186, Chile; lulloaz@udd.cl (L.U.-Z.); catapavezr@gmail.com (C.P.); anibal.riveros.o@gmail.com (A.R.); mblanco@unab.cl (F.B.-H.); 3 Centro de Estudios Postcosecha, Facultad de Ciencias Agronómicas, Universidad de Chile, Santiago 8820808, Chile; reinaldocampos@uchile.cl

**Keywords:** *NAC072*, slow ripening, auxins, gibberellins, ethylene, abscisic acid, jasmonic acid

## Abstract

Fruit development is a complex process that involves the interplay of cell division, expansion, and differentiation. As a model to study fruit development, nectarines incapable of ripening were described as slow ripening. Slow ripening fruits remained firm and exhibited no rise in CO_2_ or ethylene production rates for one month or more at 20 °C. Different studies suggest that this trait is controlled by a single gene (*NAC072*). Transcriptome analysis between normal and slow ripening fruits showed a total of 157, 269, 976, and 5.224 differentially expressed genes in each fruit developmental stage analyzed (T1, T2, T3, and T7, respectively), and no expression of *NAC072* was found in the slow ripening individuals. Using this transcriptomic information, we identified a correlation of *NAC072* with auxin-related genes and two genes associated with terpene biosynthesis. On the other hand, significant differences were observed in hormonal biosynthetic pathways during fruit development between the normal and slow ripening individuals (gibberellin, ethylene, jasmonic acid and abscisic acid). These results suggest that the absence of *NAC072* by the direct or indirect expression control of auxins or terpene-related genes prevents normal peach fruit development.

## 1. Introduction

Fruit development is a complex process that involves the interplay of cell division, expansion, and the differentiation of plant tissues, and it is carefully coordinated by many metabolic pathways controlling numerous traits such as color, aroma, size, and flavor [1,2]. Moreover, in parallel with the normal ripening (NR) process, many physiological changes occur in fruit, such as softening, fruit growth, seed development, sugar accumulation and acidity reduction, background and color skin changes, synthesis of volatile compounds, among others [2,3]. In peach, fruit ripening is characterized by presenting a double sigmoid fruit growth curve in which four stages named S1, S2, S3, and S4 can be identified. The S1 period is the first fruit growth step characterized by cell division followed by cell expansion. In S2, the fruit growth decreased significantly, and the endocarp lignification process began. Then, the second fruit growth step occurred in S3 by cell expansion, and fruit maturation was complete at the end of this period. Finally, ripening is the period corresponding to S4 [4].

Several plant hormones play an essential role in this complex maturity process temporarily and spatially synchronizing the different fruit developmental stages [1]. Auxins, gibberellins (GA), cytokinins, ethylene, jasmonic acid (JA), and abscisic acid (ABA) have been identified to be involved in different fruit stages controlling the normal fruit growth [5]. It was reported that auxins, GA, and in some cases cytokinins play crucial roles in fruits [6]. For instance, it was suggested that an auxin-mediated promotion of GA synthesis occurs after fertilization in the ovules and valves, thereby stimulating fruit growth [7]. While auxins are described by initiating fruit development and, with gibberellins maintaining fruit growth, ethylene is the senescence hormone. Ethylene evolution during fruit maturation in peach was described by [8]. This hormone was observed during the early S1 stage and in ripening (the end of S4), with the highest concentration values during ripening being positively associated with high activity levels of the enzyme 1-aminocyclopropane-1-carboxylic acid oxidase (ACO). Jasmonic acid (JA) mediates plant responses to environmental stresses such as wounding, insects, and pathogen attack [9], but it also plays a role during developmental processes, including root growth, seed germination, pollen and fruit development, and ripening [10]. On the other hand, the hormone abscisic acid (ABA) was related with the stimulation of color development and sugar accumulation during fruit ripening in peach, and associated with the modulation of the biosynthesis of ethylene and auxins by strongly affecting related gene expression during the S3/S4 developmental stages [11].

As previously mentioned, peach fruit ripening is accompanied by changes in color, aroma, size, and flavor. These changes are controlled by complex hormonal machinery allowing to development of a normal ripen fruit. For the first time, a nectarine genotype originated from Fantasia incapable of ripening and described as the slow ripening (SR) phenotype was identified [12]. Slow ripening fruit remained firm and green and exhibited no rise in CO_2_ or ethylene production rates during more than one month at 20 °C. Previous studies have suggested that this trait is controlled by a single recessive gene (sr) [13], and by comparing the seed and mesocarp development between the Fantasia and SR phenotypes, it was evident that the mesocarp development of SR individuals seems to be blocked at stage S3 [4].

Furthermore, a deletion of 26.6 kbp was identified in an F2 population from the self-pollination of Venus (V × V) associated with this trait [14], and a diagnostic marker was developed for the SR phenotype in a Belbinette and Nectalady (Bb × Nl) population [15]. Both results have the same candidate gene for the SR phenotype, described as an NAC transcription factor (Prupe.4G186800) located in chromosome 4 of the peach genome, suggestive of a correlation between the SR phenotype and the maturity date [16]. 

NAC transcription factors are a large family of structurally distinct and functionally diverse plant-specific proteins. More than one hundred NAC genes have been identified in Arabidopsis [17]. This family was associated with several functions such as plant development [18], lateral root formation and auxin signaling [19], defense [20], and abiotic stress [21,22]. However, little was reported about NAC transcriptional and post-translational regulation. In addition, it is known that NAC proteins can homo- and hetero-dimerize and interact with other transcription factors, suggesting combinatorial regulation of transcription factor activity. Hence, an important goal of NAC protein research is to determine the complexities of the NAC transcription factor network and to identify possible target genes to understand this plant transcriptional machinery [23].

Although the evidence mentioned above relates *NAC072* to the regulation of maturity date and SR phenotypes, little is known about the molecular mechanisms involved in this regulation. For this reason, this work aims to understand the molecular basis of the SR phenotype and how the absence of *NAC072* (Prupe.4G186800) acts in regulating peach fruit development using a transcriptomic approach between NR and SR individuals.

## 2. Results

### 2.1. Slow Ripening Phenotype and Candidate Gene *NAC072* Expression Profile

**Figure 1:**
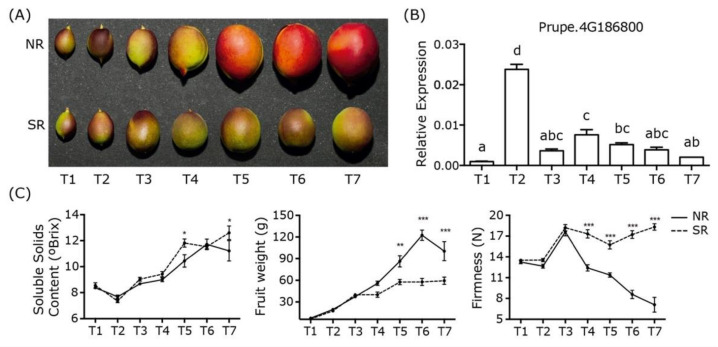
Slow ripening versus normal ripening fruit phenotypes. Fruit changes between normal and slow ripening phenotypes at different developmental stages. The evaluation stages T1, T2, T3, T4, T5, T6 and T7 represent different days after 1 September (DASF), corresponding to 37, 51, 65, 79, 99, 112 and 120 DASF, respectively. (**A**) Photographic developmental evaluation of fruit with normal (NR) and slow (SR) ripening phenotypes. (**B**) Expression profile of the candidate gene Prupe.4G186800 (*NAC072*) during normal phenotype development. Letters a-d represent significant expression differences. (**C**) Changes in soluble solid content, fruit weight and firmness between NR (continuous lines) and SR (dashed lines) phenotypes. Significant differences between NR and SR phenotypes are represented with asterisk (* *p* < 0.05; ** *p* < 0.01; *** *p* < 0.001).

The SR phenotype was recently identified as a monogenic trait incapable of ripening. Figure 1 displays a comparison between NR and SR phenotypes from T1 (37 DASF; days after 1 September) to T7 (120 DASF), representing the period between 1 week after fruit set and harvest time. SR fruit development seems to be stopped in T3 (65 DASF) and skin color changes did not develop even at fruit harvest time in T7 (Figure 1A). Also, this phenotype is still present in the tree a few months after harvest. Differences in other traits between both phenotypes include soluble solid content, fruit weight, and firmness (Figure 1C). A slight difference in soluble solid content can be observed, with a higher amount observed in the SR fruit in T5 and T7, respectively. A significant change in fruit weight starting in T5 was shown, where the NR reached approximately twice the SR weight values. In addition, for firmness, we observed that in T4, no softening process occurred in the SR phenotype compared to the softening process observed in NR fruit (Figure 1C).

In parallel, the expression profile of the SR candidate gene Prupe.4G186800 (*NAC072*) was assessed in the same evaluation periods to identify the developmental stages to perform the transcriptomic analysis. The results for the NR fruit showed negligible expression of *NAC072* in T1 followed by a peak of expression in T2 (51 DASF), that decreased immediately in T3, maintaining a low expression level until T7 (Figure 1B). The results for the SR fruit showed no expression of *NAC072* at any developmental stage. 

The transcriptomic analysis included four development stages selected based on the *NAC072* expression profile, where T2 seems to be the most critical point to understand the molecular mechanisms by which *NAC072* controls fruit ripening (Figure 1B). For this reason, T1, T2, and T3 were selected. On the other hand, T7 was selected because it is the developmental stage with significant differences between the NR and SR phenotypes, considering all information presented in Figure 1. 

### 2.2. RNA Sequencing and Bioinformatic Analysis

The selected samples and the sequencing information details are presented in Table S1. The number of reads sequenced for each sample was 46,229,240 reads, with a GC content of 45% on average and no overrepresented libraries. When the samples were filtered, between 0.6% and 0.9% of the reads were lost by quality on each library. The alignment process resulted in an average of 93.3% of the filtered reads correctly aligned against the reference genome. In summary, of the total reads sequenced for each library, 92.8% on average were correctly aligned with the reference genome, and no overrepresented libraries and contaminations were found.

**Figure 2:**
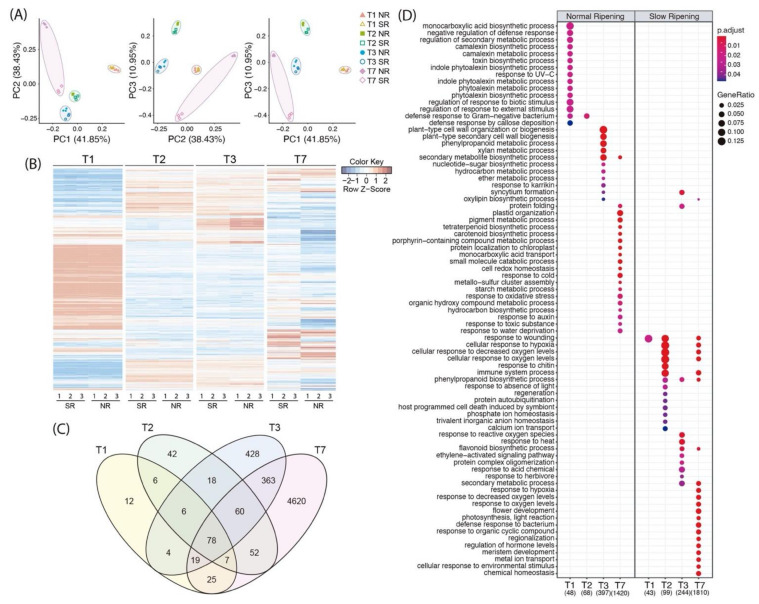
Differential expression analysis between NR and SR phenotypes. Graphical representation of differentially expressed transcripts for the four developmental stages analyzed (T1, T2, T3, T7) between the NR and SR phenotypes. (**A**) Principal component analysis using differentially expressed transcripts. The different stages are separated in colored circles of red, green, blue and purple for T1, T2, T3 and T7, respectively. The NR phenotype is represented with fullly colored figures and the SR phenotype is represented with line colored figures. (**B**) Heatmap of differentially expressed genes between NR and SR phenotypes. The red and blue lines represent genes with up and down regulated expressions, respectively. The four developmental stages are separated, and each column represents one independent biological replicate expression result. (**C**) Venn diagram representing the differentially expressed transcripts. In yellow, green, blue and purple, the differentially expressed transcripts only occurring in T1, T2, T3 and T7, respectively are represented, and the number of identified genes differentially expressed in all developmental stages are presented in grey. (**D**) Gene ontology term enrichment analysis of normal and slow ripening phenotypes in fruit development. The blue-red scale color represents the adjusted p-value and the point size represents the DE gene ratio.

A principal component analysis (PCA) was performed to observe the biological replicate distribution using the expression levels of each transcript. Figure 2A shows that the replicates of each sample presented similar behaviors and grouped close to each other. Furthermore, the four development stages analyzed are clearly separated. Two significant differences were identified, PC1 (41.85%) separated the T1 samples from the other developmental stages, and PC2 (38.43%) separated the T7 samples of NR from the SR. The T2, T3, and T7 SR samples showed a similar trend among them, and could only be separated by PC3 (10,95%). Similar results are demonstrated in Figure 2B, which uses all differential expression data to construct a heatmap. In general, the differences between T1 and the other three developmental stages were evident, as was the difference between the NR and SR T7 samples.

Furthermore, the number of differentially expressed genes (DEG) in each developmental stage were compared separately through the expression levels between NR and SR individuals in a Venn diagram (Figure 2C). A total of 157, 269, 976, and 5,224 DEG were identified between the normal and the mutant phenotypes for T1, T2, T3, and T7, respectively. The section in gray displays the 78 transcripts differentially expressed in all developmental stages. In this group of genes, the described candidate gene for SR phenotype *NAC072* is included (Figure 2C). Of these 78 genes related to the SR phenotype, three stand out for their description: one WRKY transcription factor (*WRKY35*) and two auxin-related genes (SAUR family proteins). These three genes presented with considerably higher expression values in NR individuals.

A gene ontology term enrichment analysis was carried out to understands the fruit transcriptomic differences between the normal and slow ripening phenotypes (Figure 2D). Regarding the development of normally ripening fruits, it was possible to identify high activity of cell wall remodeling enzymes in T3, accompanied by nucleotide-sugar biosynthesis. While in the T7 NR fruits, increases in the genes associated with carotene biosynthesis, auxin response, and oxidative stress were observed. When analyzing the transcriptomes of slow ripening fruits, neither cell wall remodeling nor carotene biosynthesis were observed. The stress response was mainly identified from T2 to T7 where the response to hypoxia, response to chitin, response to decreased oxygen levels, host programmed cell death, response to reactive oxygen species, and regulation of hormone levels stood out.

To identify possible candidate genes directly related to *NAC072*, genes with no expression in one condition (expression patterns like *NAC072*) were selected, resulting in 43 candidate genes. Eleven candidates seemed to be associate with the studied phenotype: one 1-aminocyclopropane-1-carboxylate synthase 1 (*ACS1*), five auxin-related transcripts (SAUR family proteins), and one cell wall remodeling enzyme described as glucosyltransferase, all of which were expressed only in the NR phenotype. Conversely, two candidate genes described as α-farnesene synthases 1 (*AFS1*), one cell number regulator, and one cell division control protein were identified, which were expressed only in the SR phenotype (Table 1). 

### 2.3. Hormonal Differences between Normal and Slow Ripening Phenotypes

**Figure 3:**
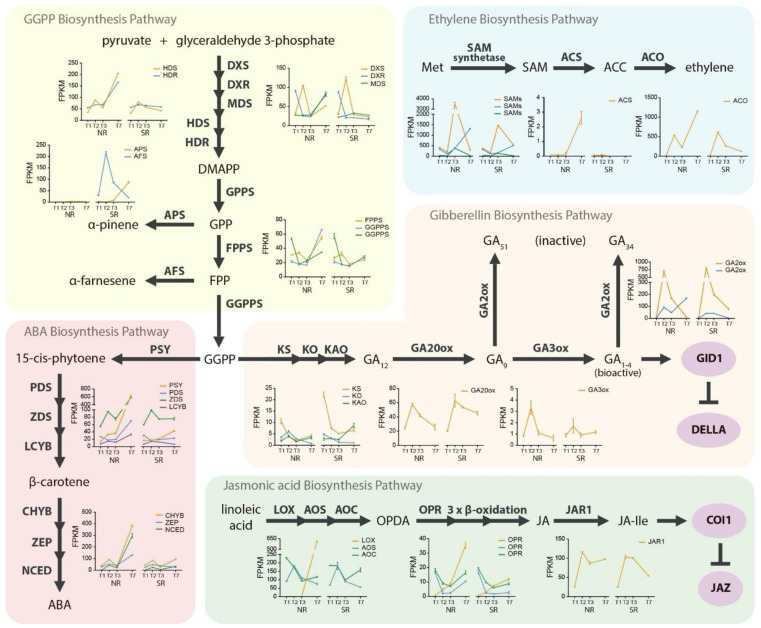
Graphical representation of hormone biosynthetic pathways related to fruit development. Schematic diagram of different hormone biosynthetic pathways related to fruit development are represented in separated colored boxes. Each transcript involved in a specific hormone biosynthetic step is in bold, and their expression profiles are represented in different graphs only for genes with differential expression identified by RNAseq with FDR < 0.05 and FC > |1|. The expression profiles of the NR and SR transcripts are represented on the left and right side of each graph, respectively. DXS, 1-deoxy-D-xylulose-5-phosphate synthase; DXR, 1-deoxy-D-xylulose-5-phosphate reductase; MDS, 2-C-methyl-D-erythritol 2,4-cyclodiphosphate synthase; HDS, hydroxymethylbutenyl 4-diphosphate synthase; HDR, hydroxymethylbutenyl 4-diphosphate reductase; DMAPP, dimethylallyl pyrophosphate; GPP, geranyl diphosphate; GPPS, GPP synthase; APS, α-pinene synthase; FPP, farnesyl diphosphate; FPPS, FPP synthase; AFS, α-farnesene synthase; GGPP, geranylgeranyl diphosphate; GGPPS, GGPP synthase; PSY, phytoene synthase; PDS, phytoene dehydrogenase; ZDS, ζ-carotene desaturase; LCYB, lycopene- β-cyclase; CHYB, β-carotene hydroxylase; ZEP, zeaxanthin epoxidase; NCED, 9-cis-epoxycarotenoid dioxygenase; KS, ent-kaurene synthase; KO, ent-kaurene oxidase; KAO, ent-kaurenoic acid oxidase; GA20ox, gibberellin 20-oxidase; GA3ox, gibberellin 3-β-dioxygenase; GA2ox, gibberellin 2-β-dioxygenase; Met, methionine; SAM, S-adenosylmethionine; ACC, 1-aminocyclopropane-1-carboxylic acid; ACS, ACC synthase; ACO, ACC oxidase; LOX, lipoxygenase; AOS, allen oxide synthase; AOC, allen oxide cyclase; OPDA, 12-oxo-phytodienoic acid; OPR, 12-oxophytodienoate reductase; JAR1, jasmonic acid-amino synthetase.

Fruit development is a complex process that is coordinated carefully by the interplay of many plant hormones. Furthermore, a normal ripening process involves the participation of these hormones in specific developmental stages. Therefore, hormone biosynthetic pathways related to fruit development were analyzed, and differences in expression profiles were observed in the genes related to ethylene, abscisic acid (ABA), gibberellins (GA), and jasmonic acid (JA) production (Figure 3). 

#### 2.3.1. Ethylene Production

It is well known that SR fruits do not produce ethylene, unlike NR fruits. Methionine is the starting substrate to produce ethylene through the activity of 3 enzymes, an S-adenosylmethionine (SAM) synthase that produce SAM, a 1-aminocyclopropane-1-carboxylic acid (ACC) synthase (ACS) that produce ACC, and finally, an ACC oxidase (ACO) that produce ethylene (Figure 3, blue section). This information was analyzed with the transcriptomic data. Thus, in the expression profiles of three SAM synthases, one *ACS* and one *ACO*, the most important differences were observed in the ACS and ACO expression levels. In NR siblings, *ACS* showed a unique peak of expression during ripening (T7), and no expression of this gene was observed in SR individuals. On the other hand, unlike the expression pattern of *ACS*, two peaks were observed for normal *ACO* expression, one in T2 without differences between NR and SR individuals, and the other at ripening (T7). *ACO* was absent in SR siblings with a differential expression of this gene in T7 with a fold change of 3.2 (Figure 3). In summary, differences in *ACS* and *ACO* expression patterns were observed only in the last developmental stage (T7), probably associated indirectly with the absence of *NAC072* and supporting the decreased ethylene production.

#### 2.3.2. Geranylgeranyl Diphosphate Biosynthesis, Precursor of ABA and GA Production

Abscisic acid and gibberellin biosynthesis pathways start with the same precursor, geranylgeranyl diphosphate (GGPP), produced by the metabolism of pyruvate and glyceraldehyde 3-phosphate (Figure 3, yellow section). Our results showed differences in all enzymes that belong to the canonical GGPP biosynthetic pathway in T7, and we observed a lower abundance of these transcripts in SR fruit. However, no differences were observed in the early developmental stages. In addition, two other candidates were found: the α-farnesene and α-pinene synthases (*AFS* and *APS*, respectively). The first gene was found with an early differential expression in T2 redirecting FPP to promote α-farnesene biosynthesis. The second gene was found differentially expressed in T7, redirecting GPP to promote α-pinene biosynthesis (Figure 3). Both transcripts were expressed only in SR siblings, probably decreasing the GGPP production in the SR phenotype. Thus, the transcriptomic data suggest lower GGPP production in SR siblings during ripening by the canonical GGPP biosynthesis pathway, ans also FPP and GPP redirection to terpene biosynthesis, further reducing GGPP production in these individuals in the early developmental stages. 

The expression patterns of the transcripts belonging to the ABA biosynthesis pathway (Figure 3, red section) resulted in a similar expression profiles between NR and SR individuals in T1, T2, and T3. However, in T7, a higher abundance of all transcripts related to the ABA biosynthesis pathway was observed in NR fruit, suggesting a lower ABA production on SR individuals only in T7, as previously mentioned for the GGPP.

Even though we did not observe the exact behavior of GGPP and ABA biosynthesis in GA production (Figure 3, orange section), a significant difference was observed in the expression profile of one *GA3ox* in T2 with a lower expression level in the SR siblings. The candidate genes *NAC072* (Figure 1B) and AFS (Figure 3 yellow section) had same point of highest expression. This enzyme produces bioactive GA and is a critical enzyme in GA activity and regulation, suggesting lower bioactive GA production in SR siblings.

In summary, SR individuals presented lower production of ABA and GA during ripening, mainly because there was a decrease in GGPP production (a precursor of both hormones) in T7. In addition, the lower GGPP production in T7 was accompanied by a lower transcript abundance of ABA biosynthetic-related genes. Moreover, a lower *GA3ox* expression level was identified in T2, suggesting lower bioactive GA activity in early fruit developmental stages and a more direct relationship between *NAC072*, *AFS* and *GA3ox*. 

#### 2.3.3. JA Production

Linoleic acid is converted to 12-oxo-phytodienoic acid (OPDA) through the enzymes lipoxygenase (LOX), allene oxide synthase (AOS) and allene oxide cyclase (AOC), then is transformed through the activity of the enzyme 12-oxophytodienoate reductase (OPR) and a series of β-oxidations to form jasmonic acid (Figure 3, green section). The results obtained showed a higher abundance of one *LOX* and three *OPR* in normal ripening fruit, suggesting a higher JA production only in T7. The *LOX* described here had an FC = 9.3 with 613.0 FPKM in the NR siblings, and low expression in the SR individuals (FPKM < 1.0). Although other *LOX* genes were overexpressed in the SR siblings, their expression levels were considerably lower than those mentioned above (data not shown). On the other hand, there were six genes described as *OPR2* in the peach genome. Three of them displayed differences in their expression levels at T7 (Figure 3, green section), suggesting higher OPDA accumulation and lower JA biosynthesis in SR individuals.

Finally, JA needs to be conjugated into JA-Ile through the activity of the enzyme jasmonic acid-amino synthetase (JAR1) to perform its biological function. Our results showed that *JAR1* abundance increased significantly in T2 and remined high in NR fruit in T7 (Figure 3, green section). The SR fruit presented similar *JAR1* abundance behavior in T1, T2, and T3, but then showed a significant decrease in T7. These results suggest that SR fruit displays less JA production than regular ripening fruit. Thus, we hypothesize that there might also be less JA conjugation to JA-Ile.

### 2.4. RNAseq Validation by qPCR

**Figure 4:**
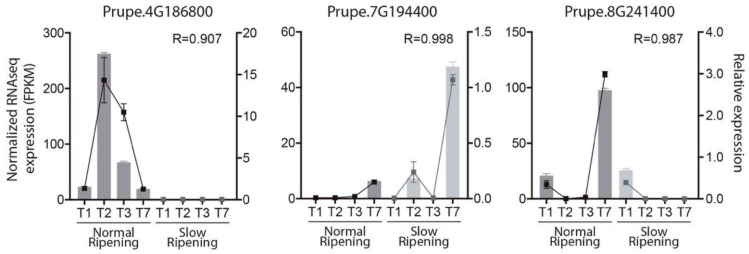
Validation of RNAseq results by qPCR. Comparison between normal and slow ripening expression profiles determined by RNAseq and qPCR of three differentially expressed candidate genes are displayed. The left axis represents the expression profile, in FPKM, of genes measured by RNAseq (bars). The right axis presents the relative expression profiles of genes measured by qPCR (lines). A Pearson correlation was calculated between the RNAseq and qPCR expression data and presented in each graph as the R value.

To validate the candidate genes obtained using the RNAseq information, the expression profiles of three genes were measured by qPCR in the NR and SR individuals. Figure 4 shows the relative expressions of the *NAC072* (Prupe.4G186800), *ERF017* (Prupe.7G194400) and *FAF* (Prupe.8G241400) genes using RPII as control and compared with the expression levels (FPKM) obtained by RNAseq analysis. For *NAC072*, a peak of expression in NR individuals was in T2 and no expression values were found in SR individuals. For *ERF017*, higher expression values were expected for T7 in the SR phenotypes. Finally, for *FAF*, two peaks of expression in T2 and T7 for NR siblings were expected, but for the SR siblings the only expression values were found in T2. In summary, similar results were obtained by qPCR analysis for all genes analyzed. Thus, the the RNAseq expression results obtained were validated.

## 3. Discussion

### 3.1. Phenotyping

The SR phenotype was reported for the first time in 1987 in slow-ripening nectarines derived from the cultivar Fantasia that mature approximately 2.5 months after commercially harvested, normal ripening Fantasia, and this slow ripening phenotype did not present flesh color and firmness changes [12]. These slow ripening nectarines failed to produce normal ethylene levels for at least one month after harvest and their ethylene production peaks were significantly reduced [24]. Conversely, the SR siblings displayed a more significant accumulation of soluble solids and less titratable acidity than regular ripening fruit [12]. Similar results were observed in the V×V population (Figure 1). Significant differences were obtained in size, color, and firmness between the NR and SR phenotypes. These changes were identified in the S2 development stage between T3 and T4 (65–87 DASF), the period in which the process of endocarp lignification (pit hardening) begins, and fruit growth was consequently reduced [4]. At S3, no fruit growth was observed in the SR siblings, suggesting fruit development stagnation in stage S2.

The expression profile of the candidate gene *NAC072* was analyzed (Figure 1). One peak of expression was observed during fruit development at T2 (51 DASF). This period was identified as the transition from S1 (first fruit growth period) to S2 (endocarp lignification), and is also the period when the embryo starts to develop the seed [4]. Furthermore, as mentioned before, the differences between the NR and SR phenotypes were observed between T3 and T4. For these reasons, it seems possible that *NAC072* id involved in fruit, seed, or embryo development, and its absence may cause the slow ripening phenotype.

### 3.2. Sequencing Results and Differential Expression Analysis 

All developmental stages of the NR and SR phenotypes were compared at the transcriptomic level. Even though significant differences between all developmental stages in NR phenotypes were expected, the transcriptomic results showed that the fruit mesocarp did not display such large differences between T2 and T3 (Figure 2A,B). These results were probably because this period corresponds to a reduction in fruit growth and seed development, and the beginning of lignification [4]. In the case of the transcriptomic information of SR, in T1, T2, and T3 the SR information was similar to that of NR, but in T7 significant differences were observed between NR and SR, where T7 SR samples seemed to be more similar to T2 and T3, respectively. These results suggest a stop in SR development between the T2 and T3 developmental stages (Figure 2A). It is possible that the absence of *NAC072* triggers an alteration in seed development or the lignification process; however, a detailed analysis of seed development could help us better understand why the slow ripening fruits stop their growth and how *NAC072* is involved in this phenotype.

Considering the differences between NR and SR, a total of 78 genes were differentially expressed in all developmental stages (Figure 2C), among them was *NAC072*. Four of these genes seemed to have a correlation with the slow ripening phenotype either due to their function or the metabolic pathway to which they belong. These genes were described as an α-farnesene synthase, the transcription factor WRKY, and two auxin-related genes described as small auxin up RNA (SAUR), thus indicating a possible correlation with the *NAC072* regulation pathway. Even though we expected to find other genes with the same expression pattern as *NAC072* in this group, none of them presented exactly the same expression pattern.

On the other hand, it is possible to identify a developmental stagnation of the slow ripening fruits that begins to be noticed in T3 (Figure 2D), when it is not possible to observe the enriched gene ontology terms of the cell wall remodeling enzymes. Neither gene ontology terms are observed for pigmentation or carotene synthesis in T7 for the slow ripening individuals, which agrees with the phenotypes observed in Figure 1A, where the normal ripening phenotype develops a change in pigmentation and fruit size accompanied by the softening process (Figure 1C). In contrast, the SR phenotype seems to be stopped in the middle of fruit development.

### 3.3. *NAC072* and Direct Possible Target Genes 

*NAC072* presented a peak of expression in T2 (Figure 1), probably affecting the S1/S2 transition or altering the seed/embryo development. We know that NR and SR individuals are similar in phenotype in T1 and T2 (Figure 1), and considering the transcriptomic information, no significant differences were observed in T1 and T2 (Figure 2). These results reinforce the hypothesis that *NAC072* is responsible for the slow ripening phenotype. Similar to *NAC072*, another 51 genes with descriptions displayed expression only in the normal ripening phenotype. Among them, five were described as SAUR family proteins and one as a 1-aminocyclopropane-1-carboxylate synthase 1 (*ACS1*), associating *NAC072* with auxin and the ethylene signaling pathways. None of them presented an expression pattern like *NAC072* and for the most part they were genes that, in normal ripening fruits, are expressed only in late stages of development (Table 1), suggesting the indirect *NAC072* regulation on these genes. On the other hand, unlike *NAC072*, 64 genes with descriptions were only expressed in slow ripening fruit. Of them, two α-farnesene synthases 1 (*AFS1*) and two genes related to cell number regulation were distinguished, where the two AFS (terpene biosynthesis-related proteins) showed expression patterns like that of *NAC072* (Table 1). 

The plant hormone auxin, or indole-3-acetic acid (IAA) is responsible for various aspects of plant development [25], including organ initiation from the shoot apical meristem (SAM) and flower/fruit development [26]. The auxin response is mediated by auxin response factors (ARF) that may activate or repress the expression of early auxin-responsive genes like the small auxin up RNA (SAUR) genes mentioned before [25]. In general, these SAUR family genes have an unknown function, but some of them have been associated with cell expansion on the hypocotyl and leaves (SAUR19) [27], auxin synthesis regulation (SAUR39) [28], and leaf senescence (SAUR36) [29]. In fruit, a crucial role of endogenous IAA was reported as controlling the onset of ripening in fleshy fruit and regulate fruit growth and development together with GAs [7]. On the other hand, an association between *NAC072* and ethylene was identified through the differential expression of the gene *ACS1* between the normal and slow ripening individuals. *ACS1* was identified as a critical enzyme in ethylene biosynthesis [30], using S-adenosylmethionine (AdoMet) to produce 1-aminocyclopropane-1-carboxylate (ACC). Therefore, its regulation at the transcriptional level is an important factor regulating ethylene production in response to different stimuli. ACS protein stability also plays a significant role in controlling ethylene biosynthesis [31]. However, as *ACS1* presents its expression only in late stages of development (Figure 3, blue section), we believe that it could be a consequence of the loss of the *NAC072* gene in earlier stages of fruit development, and not a direct regulation between *NAC072* and *ACS1*.

Moreover, α-farnesene synthase 1 (*AFS1*) is an enzyme that uses farnesyl diphosphate (FPP) to produce α-farnesene [32]. This α-farnesene is a sesquiterpene described as a lepidopteran attractant and an oviposition inducer [33]. It was reported to be produced during the storage of apple fruit and its oxidation is hypothesized to be the causal agent of superficial scald [34]. No correlations have been reported between *AFS1* and the slow ripening phenotype, with respect to either maturity date *NAC072*. However, the precursor FPP, used to produce α-farnesene, is also used to produce geranylgeranyl diphosphate (GGPP), a key substrate for ABA and GA biosynthesis, two plant hormone biosynthesis [33]. Thus, our results suggest a possible correlation between the expression of *AFS1* in SR individuals and ABA or GA production, but the regulation mechanisms involved remain unknown.

### 3.4. Ethylene Production Results

The plant hormone ethylene plays a key role in climacteric fruit ripening. Studies of ethylene signaling components have revealed a linear transduction pathway leading to the activation of ethylene response factors [35]. In NR fruit, ethylene is produced in the last fruit developmental stages, beginning approximately at 80 DASF (S4) [8], participating directly in fruit ripening, and it is biosynthesized by the action of three enzymes, S-adenosylmethionine (SAM) synthase, 1-aminocyclopropane-1-carboxylate synthase (ACS) and 1-aminocyclopropane-1-carboxylate oxidase (ACO). The last two enzymes (ACS and ACO) are critical to ethylene biosynthesis [36]. The normal expression patterns of these genes are shown in Figure 3 (blue section), where *ACS* displayed only one expression peak in T7, and *ACO* displayed two expression peaks in T2 and T7, respectively. By comparing the expression patterns of *ACS* and *ACO* with ethylene production (only observed in T7), *ACS* seems to be the limiting enzyme in ethylene production. Similar results were reported when comparing the ACS and ACO enzyme activity with the fruits’ 1-aminocyclopropane-1-carboxylate (ACC) content and ethylene production [8]. 

No ethylene production was identified in SR individuals [13]. This observation was validated in the transcriptomic analysis, where no *ACS* RNA accumulation was found in T7; this enzyme uses ado-met to produce ACC in normal conditions (Figure 3, blue section). These results suggest a lower accumulation of ACC in slow ripening individuals as there is no expression of the *ACS* gene. On the other hand, the *ACO* transcript accumulation was analyzed; this enzyme participated in converting ACC to ethylene [37]. In normal conditions, this enzyme has two peaks of expression in T2 and T7, but in SR individuals, transcript accumulation was found in only T2. 

In summary, the slow ripening phenotype displayed no *ACS* and *ACO* transcript accumulation in T7 (ripening period), and a lower ACC accumulation in the slow ripening siblings is suggested. These backgrounds could explain why no ethylene production was found in the slow ripening siblings during the ripening stage. Although we suspect an indirect correlation between the absence of *NAC072* and ACS transcript accumulation, the mechanism by which this regulation is carried out is not yet clear. Another phenotype of peach tree similar to the slow ripening phenotype that also does not produce ethylene is stony hard; a candidate gene for this phenotype is *YUC11* which was described as regulating the synthesis of auxins and ethylene, having the same expression pattern as *ACS1* (both genes are absent in the stony hard phenotype) [38]. However, unlike slow ripening fruits, fruits with the stony hard phenotype respond to exogenous applications of ethylene (data not shown), suggesting that slow ripening is independent of the stony hard phenotype.

### 3.5. Gibberellin Production Results

Gibberellins are diterpene plant hormones described as growth regulators with a key role in fruit development. The major bioactive GAs are GA1 and GA4 [39]. The biosynthesis of bioactive GAs and their deactivation pathways are tightly regulated processes. In this sense, as we show in Figure 3, the enzyme GA20ox is an intermediate in the GA biosynthesis pathway promoting the bioactive GA synthesis together with GA3ox. In contrast, GA2ox antagonizes GA activity by deactivating GAs [40].

The results of this research suggest that bioactive GA production in SR individuals was significantly reduced. Three antecedents that support this statement are: (i) all genes related to GGPP production (a precursor of GA biosynthesis pathway) at the ripening stage decreased their accumulation, resulting in less available GGPP to produce GAs in the SR phenotype; (ii) two α-farnesene synthases expressed only in SR individuals were found, which use farnesyl diphosphate (an intermediate in GGPP biosynthesis) to produce α-farnesene, decreasing GGPP biosynthesis and therefore also GA production, and (iii) a differential transcript accumulation of *GA3ox*, a key enzyme in bioactive GA production was observed, with less abundance of this gene in the SR phenotype in early stages of fruit development, suggesting that there is even less bioactive GA biosynthesis in SR fruit.

Previous studies have reported that different plant hormones are implicated in fruit and seed development [41]. It is well known that in fleshy fruit, the presence of seeds must promote fruit growth. In the absence of seeds, fruit growth can be stimulated with the exogenous application of auxins and gibberellins [1], suggesting that seed development is necessary for auxin/gibberellin production and fruit growth. It was previously reported that auxins induce GA production in pea fruit (*Pisum sativum*), increasing *GA3ox* transcript accumulation and decreasing GA2ox transcript levels [39,42]. Our results suggest that the SR phenotype may be related to changes in seed development and auxin–gibberellin regulation in early fruit developmental stages (T2). Exogenous applications of these hormones in early stages of development could help determine the role that auxins and gibberellins have in fruit development, seed development, and fruit transition from stage S1 to S2.

### 3.6. Abscisic Acid and Jasmonic Acid Production Results

The phytohormone abscisic acid (ABA) is an isoprenoid [43] with reported roles in embryogenesis and seed maturation [44], seed dormancy and germination [45], and adaptation to abiotic stress [46,47]. Previous studies have reported a correlation between the ABA and GA hormonal pathways during seed development. During late embryogenesis, ABA promoted seed germination, blocking the embryo growth by counteracting the function of GA [48]. Transcript accumulation differences were observed in all genes related to the ABA biosynthetic pathway at the ripening stage (T7). These results suggest less ABA production in SR individuals at T7. In addition, since β-carotene is an intermediate substrate in the ABA biosynthetic pathway, our results suggest that there may also be a reduction of the accumulation of β-carotene in SR individuals. The molecular mechanism by which the absence of *NAC072* at T2 affects ABA production at T7 remains unclear. 

On the other hand, jasmonic acid (JA) is an endogenous plant hormone responsible for the plant response to biotic and abiotic stress [49]. JA is also involved in fruit ripening, pollen survival, root growth, and plant response to injury [50,51]. JA is derived from the octadecanoid pathway by forming a 12-oxophytodienoic acid (OPDA), a precursor of JA [52,53]. Previous research reported that OPDA and JA act independently to promote different plant responses [54]. The enzymes described as 12-oxophytodienoate reductase (OPR) have a key role in regulating OPDA/JA accumulation by using OPDA to promote JA biosynthesis [55]. In this study, the expression differences in several OPRs at ripening were identified between the NR and SR phenotypes. These results suggest a differential OPDA or JA accumulation and differentially signaling responses between the NR and SR phenotypes, but experiments such as exogenous applications of OPDA or JA in slow ripening individuals are necessary to confirm this hypothesis.

The results obtained in this research suggest lower ABA and JA production in SR individuals only in late stages of fruit development (T7; ripening) supported by the lesser transcript accumulation of genes related to these hormone biosynthetic pathways (Figure 3). Therefore, it is possible that the differences related to ethylene, ABA, and JA in T7 do not have a direct correlation with the candidate gene *NAC072*. This observation was derived; since *NAC072* showed a peak of expression in T2, we believe that it causes an arrest of the normal development of the peach fruit. Considering that ethylene, ABA and JA are hormones with an essential function in normal fruit ripening, and that they have been widely studied in the later stages of its development, it is possible that the observed hormonal differences were a consequence of the effect of *NAC072* in peach development, and not a direct regulation of *NAC072*.

## 4. Materials and Methods

### 4.1. Vegetal Material and Phenotyping

An F2 population with 151 siblings previously used in fruit quality trait studies [14] was assessed to perform transcriptomic analysis between normal and slow ripening individuals. This population was obtained from the self-pollination of the nectarine cultivar Venus (*Prunus persica* (L.) Batsch cv. Venus). The cultivar Venus was obtained from the intra-specific cross between Stark Red Gold and Flamekist at the INIA-Rayentué facilities (VI Region, Rengo, Chile) and no permissions were necessary to collect plant material. This cultivar produces freestone melting yellow-fleshed nectarines. The Venus × Venus population (V × V) consists of 6-year-old trees grown on G × N rootstock in an experimental orchard located at 34°24′ S latitude and 70°50′ W longitude (INIA-Rayentué). 

Physiological fruit parameters and calculated averages for normal and slow ripening individuals were measured using nine fruit at seven fruit developmental stages determined as a number of days after 1 September (DASF). The measured dates were 37, 51, 65, 87, 99, 112, and 120 DASF for T1, T2, T3, T4, T5, T6, and T7, respectively. Photographic capture of the analyzed fruits was made using a static camera with the same light and photographic parameters, and then the weight, flesh firmness, and soluble solids content were measured. Two siblings were selected to obtain fruit material for transcriptomic analysis: one early ripening individuals and one slow ripening individual. Three replicates were selected for each individual at each analyzed developmental stage (T1, T2, T3, and T7) and collected to perform fruit RNA extractions and transcriptomic analysis (2 selected individuals × 3 replicates × 4 developmental stages = 24 samples).

### 4.2. RNA Extraction, Quantification and Quality Control

Total RNA of 24 samples was extracted from 100 mg of fruit flesh using a mortar and pestle along with the SpectrumTM plant total RNA kit, following the manufacturer’s instructions (Sigma Aldrich, Saint Louis, MO, USA). The quantification was performed using a Qubit^®^ 2.0 fluorometer and a QubitTM RNA BR assay kit (Thermo Fisher Scientific, Waltham, MA, USA) according to the manufacturer’s instructions. The quality control process of each RNA sample extracted was made using a Fragment Analyzer™ automated CE system (Analytical Advanced Technologies, Ames, IA, USA), 0.1–0.8 μg of total RNA were analyzed using a Standard Sensitivity RNA analysis kit (Advanced Analytical Technologies) following the manufacturer’s recommendations, and finally, ProSize 2.0 software (Analytical Advanced Technologies) was used to determine the RNA quality, considering an RQN value of 8.0 as useful for library construction and sequencing.

### 4.3. Library Construction and RNA Sequencing

The indexed libraries were built with a TruSeq^®^ RNA Library Prep Kit v2 (Illumina Inc., San Diego, CA, USA) using 1 μg of isolated RNA. They were validated by capillary electrophoresis using a Fragment Analyzer™ Automated CE System with the Standard Sensitivity NGS Analysis Kit (Advanced Analytical Technologies), followed by quantification using qPCR with a Library Quantification Complete Kit Illumina/Universal (Kapa Biosystems, Wilmington, MA, USA) in an Eco™ thermocycler (Illumina Inc.) according to manufacturer’s instructions. Validated libraries were sequenced in a HiSeq2500 with Macrogen’s service.

### 4.4. Differential Expression Analysis

The sequenced data were analyzed using FastQC software. Adapters were removed from all samples and filtered by quality (Q > 20.0) using Flexbar software. Filtered read alignments were made using RSEM software, following the developer’s recommendations [56]. An RSEM reference was made using the Prunus persica v2.0.a1 reference genome [57] by the rsem-prepare-reference script with the -gtf option to add the gene annotation file. Then, read abundance estimation for each sample was performed by the rsem-calculate-expression script using the RSEM reference previously obtained. Differential expression analysis was performed using the Bioconductor package EdgeR (FDR < 0.05; FC > |1|), following the developer instructions [58]. 

### 4.5. RNAseq Validation by qPCR

Transcript levels were analyzed by qPCR, for which 1 μg of total RNA was treated with DNase I (Thermo Fischer Scientific, Waltham, MA, USA) to eliminate gDNA contamination. The Superscript II RT system (Invitrogen, Carlsbad, CA, USA) was used for complementary DNA synthesis, according to the manufacturer’s instructions. Levels of transcripts were quantified for six selected DEGs of interest using peach fruit flesh of normal and slow ripening individuals. Every reaction was performed on an Eco system (Illumina Inc.) with Evagreen mix (Biotium, Fremont, CA, USA) and specific primers.

Three biological replicates and three technical replicates were used for each gene, and RPII was used as a control [59]. The PCR program was (i) enzyme activation at 95 °C for 10 min, with 40 cycles of (ii) 95 °C for 15 s, annealing for 15 s, and 72 °C for 15 s. After every PCR, a melting curve was generated from 55 to 95 °C. Finally, the data were analyzed with GraphPad Prism 7 (GraphPad Software, La Jolla, CA, USA), and standard error was used for the biological and technical replicates. To determine the correlation between RNAseq and qPCR expression results, a Pearson correlation coefficient was calculated for each gene analyzed.

## 5. Conclusions

This study identified three regulation levels in the SR phenotype: (i) an early auxin signaling alteration in T2, considering the identification of five auxin-related genes with no expression values in SR individuals like *NAC072*; (ii) a *GA3ox* transcript accumulation in SR individuals smaller than in NR individuals. Moreover, two α-farnesene synthases expressed only in the SR siblings suggest that there was a lower production of bioactive GA in the early stages of fruit development, and (iii) in the late fruit developmental stage (T7) of SR individuals, probably as a consequence of the fruit developmental alteration in T2, less transcript accumulation of enzymes related to ethylene, ABA and JA biosynthetic pathways were observed, suggesting a hormone production misregulation associated with the fruit ripening process. 

Moreover, we hypothesize that the function of *NAC072* was associated with seed development, considering that any transcript with a similar expression pattern to *NAC072* was identified in the fruit flesh transcriptome assay; the *NAC072* peak expression value coincided with the beginning of seed development; and *NAC072* was associated with auxin-related genes and previous studies have reported that the seeds are necessary for normal auxin signaling. 

In summary, the molecular mechanisms underlying the slow ripening phenotype might begin with the *NAC072* function associated with the seed development altering the auxin signaling, followed by the regulation of the GA biosynthesis pathway decreasing bioactive GA and fruit growth, and might be related to the production of ethylene, ABA and JA during ripening as a consequence of fruit growth alteration in the early stages of fruit development. Further proposed studies include a seed transcriptomic approach to clarify the role of *NAC072* during seed development, and hormonal measurement of auxins and gibberellins at different fruit developmental stages to identify the interplay between these two hormones during fruit growth.

## Figures and Tables

**Table 1:** *NAC072* candidate genes related to gene expression profiles.

GeneName	Slow Ripening *				Normal Ripening *				Description
	T1	T2	T3	T7	T1	T2	T3	T7	
Prupe.4G186800	-	-	-	-	22.8	251.6	64.3	18.9	*NAC072*
Prupe.2G176900	-	-	-	-	0.1	0.0	0.1	2.4	*ACS1*
Prupe.4G126600	26.4	183.7	72.7	15.3	-	-	-	-	*AFS1*
Prupe.4G126400	2.6	15.9	7.2	1.2	-	-	-	-	*AFS1*
Prupe.8G080700	-	-	-	-	15.1	5.7	1.7	39.7	SAUR family protein
Prupe.2G085100	-	-	-	-	0.0	0.0	0.0	30.6	Glucosyl transferase
Prupe.8G081800	-	-	-	-	0.0	0.0	0.0	1.9	SAUR family protein
Prupe.8G081200	-	-	-	-	0.0	0.0	0.0	1.9	SAUR family protein
Prupe.1G043300	1.1	0.0	0.5	1.1	-	-	-	-	Cell number regulator
Prupe.4G237600	0.4	0.2	0.5	0.1	-	-	-	-	Cell division control
Prupe.8G081300	-	-	-	-	0.0	0.0	0.0	1.0	SAUR family protein
Prupe.1G442200	-	-	-	-	0.0	0.0	0.0	0.7	SAUR family protein
Prupe.8G079700	-	-	-	-	0.1	0.0	0.0	0.4	*GA20ox3*

* Normalized expression represented in FPKM values. No gene expression values in one condition are represented by hyphens (-).

## Data Availability

The datasets generated and analyzed during the current study are available in the National Center for Biotechnology Information (NCBI) repository, PRJNA730315. https://www.ncbi.nlm.nih.gov/bioproject/PRJNA730315 (accessed on 1 October 2021).

## Supplementary Materials

The following are available online at https://www.mdpi.com/article/10.3390/plants10112380/s1, Table S1: Sequenced, filtered and aligned summary metrics.
